# Flaviviruses induce ER-specific remodelling of protein synthesis

**DOI:** 10.1371/journal.ppat.1012766

**Published:** 2024-12-02

**Authors:** Ho Him Wong, Dorian Richard Kenneth Crudgington, Lewis Siu, Sumana Sanyal

**Affiliations:** 1 HKU-Pasteur Research Pole, School of Public Health, Li Ka Shing Faculty of Medicine, The University of Hong Kong, Hong Kong SAR; 2 Sir William Dunn School of Pathology, South Parks Road, University of Oxford, Oxford, United Kingdom; Leiden University Medical Center: Leids Universitair Medisch Centrum, NETHERLANDS, KINGDOM OF THE

## Abstract

Flaviviruses orchestrate a unique remodelling of the endoplasmic reticulum (ER) to facilitate translation and processing of their polyprotein, giving rise to virus replication compartments. While the signal recognition particle (SRP)-dependent pathway is the canonical route for ER-targeting of nascent cellular membrane proteins, it is unknown whether flaviviruses rely on this mechanism. Here we show that Zika virus bypasses the SRP receptor via extensive interactions between the viral non-structural proteins and the host translational machinery. Remarkably, Zika virus appears to maintain ER-localised translation via NS3-SRP54 interaction instead, unlike other viruses such as influenza. Viral proteins engage SRP54 and the translocon, selectively enriching for factors supporting membrane expansion and lipid metabolism while excluding RNA binding and antiviral stress granule proteins. Our findings reveal a sophisticated viral strategy to rewire host protein synthesis pathways and create a replication-favourable subcellular niche, providing insights into viral adaptation.

## Author summary

Zika virus, like other flaviviruses, hijacks the protein-making machinery of host cells to replicate. Typically, cells use a complex system called the signal recognition particle (SRP) pathway to properly insert newly made proteins into cellular membranes. This study reveals that Zika virus has evolved a clever strategy to bypass part of this system. While the virus still needs some components of the SRP pathway, it can skip a key step involving the SRP receptors. Instead, Zika’s own proteins interact directly with parts of the cellular machinery, allowing efficient production of viral proteins at cellular membranes. This unique approach gives Zika virus an advantage, enabling it to rapidly produce the proteins it needs for replication, potentially avoiding some cellular quality control mechanisms. Furthermore, this viral strategy alters which host proteins are made, favouring those that support virus replication and suppressing those involved in antiviral responses. These findings provide insights into the ways in which viruses adapt to and exploit their hosts.

## Introduction

Zika virus (ZIKV), a member of the *Flaviviridae* family, became a major global health concern following the 2015 outbreak and its association with congenital neurological disorders such as microcephaly [1]. While ZIKV is enzootic in non-human primates [2,3], outbreaks stem from transmission via mosquito bites [4,5], with reported cases of direct human-to-human transmission [6,7].

ZIKV is an enveloped virus, ~50nm in diameter. Its genome comprises a single-stranded positive-sense RNA lacking a 3’ poly-A tail. Highly structured untranslated regions (UTRs) at the 5’ and 3’ termini interact with the viral non-structural proteins to promote replication [8]. The viral RNA encodes a single polyprotein precursor cleaved by host and viral proteases into 3 structural and 7 non-structural (NS) proteins. The structural proteins–Capsid (C), pre-Membrane (prM) and Envelope (E)–facilitate virion assembly and entry via receptor binding and membrane fusion [9,10]. The non-structural proteins induce extensive reorganisation of the host endoplasmic reticulum (ER) membrane and associated lipid droplets to establish replication compartments containing the necessary host and viral factors for replication and assembly [11–14]. The replication compartments contain narrow pores connecting the cytosol and serve as ‘factories’ supporting viral genomic replication and packaging into progeny virions [15,16].

For proper folding, cellular membrane proteins requires insertion of hydrophobic transmembrane domains (TMDs) into the ER membrane as they emerge from ribosomes, preventing aggregation in the aqueous cytosol [17]. While some TMDs, particularly those proximal to the C-terminus in tail-anchored proteins, can insert independently of the translocon via one of the three human Oxa1 homologs–the TRC40, TMCO1 or EMC complexes [18]–the majority depend on the Sec61 translocon for accurate integration into the ER membrane. The Sec61 translocon forms an aqueous channel at the ER membrane for TMD transfer and integration.

Accurate topology of TMDs relies on various targeting mechanisms that deliver the ribosome-nascent chain complex (RNC) to Sec61. Newly synthesised TMDs are targeted co-translationally by the signal recognition particle (SRP) to the Sec61 translocon [19,20]. Alternatively, post-translational targeting pathways, such as the SRP-independent (SND) or the chaperone-mediated pathways, deliver TMDs to the Sec62/Sec63 translocon [21]. Considering the multi-spanning nature of the flaviviral polyprotein, precise insertion of the first TMD is expected to facilitate accurate topology of downstream TMDs [22,23].

Flaviviruses are predicted to exploit the co-translational pathway for polyprotein biogenesis via the SRP pathway, which comprises evolutionarily conserved components in prokaryotes and eukaryotes [24,25]. In eukaryotes, the cytosolic SRP complex contains 6 subunits—SRP9, 14, 19, 54, 68 and 72 –assembled on a 7S RNA scaffold. The SRP receptor (SR) is a heterodimer of ER-localised SRα and SRβ that receives the RNC-bound SRP. Both SRα and SRβ as well as SRP54 are GTPases, whose binding and hydrolysis of GTP regulates this process [26,27]. During nascent chain synthesis, the SRP54 subunit recognises and binds hydrophobic signal sequences as they emerge from the ribosomal exit tunnel, forming an SRP-RNC complex that stalls translation. The SRP-RNC interacts with the ER-localised SR, enabling GTP loading onto SRP54 and SRα that facilitates RNC transfer to Sec61, restoring translation. Subsequent GTP hydrolysis triggers SRP-SR dissociation from the translocon [26].

Previous reports have highlighted that flaviviruses exploit numerous host translation factors for virus production [10]. Host signal peptidases are essential for cleavage of the polyprotein in multiple flaviviruses [28,29]. Additional factors such as oligosaccharide transferase complex (OST) and the translocon-associated protein complex (TRAP) facilitate virus propagation [28,29], while the ribosome binding protein RRBP1 stabilises viral RNAs and enable translation [30]. The EMC assists appropriate membrane insertion of dengue and Zika virus NS4A/NS4B proteins [31,32]. Lastly, upregulation of the SRP components SRP54, SRP72 and SRα was reported in a study using DENV2 infected A549 cells [33]. Interestingly however, while depletion of select SRP subunits such as SRP9, 14 and 72 restricted replication of some flaviviruses [28,29,34], the SRP receptors SRα and SRβ have not emerged as critical host dependency factors from unbiased screens [28–30,34]. Furthermore, SRβ was found to be excluded from the sites of Zika virus NS4A localisation at the ER [35]. This has generated a gap in our understanding of a key step in flavivirus biology, i.e., how a nascent flavivirus polyprotein is targeted to the ER membrane.

Here we demonstrate that Zika virus leverages the SRP pathway for biogenesis, but remarkably uncouples the SRP-SR interaction via its non-structural proteins, NS3 in particular. SR-depletion failed to attenuate Zika virus protein synthesis and virion production, unlike influenza virus, which was significantly inhibited. Our data suggest a model where the nascent viral protein uses SR-independent and viral NS3-assisted ER targeting, where recruitment of the translocon, ribosome, and SRP by viral non-structural proteins bolsters selected host and viral protein synthesis in a SR-independent manner. Our findings highlight a unique viral strategy to co-opt the host translation machinery and ensure efficient replication.

## Results

### ZIKV polyprotein is inserted via the SRP- and Sec61 translocon-dependent pathways

**Fig 1:**
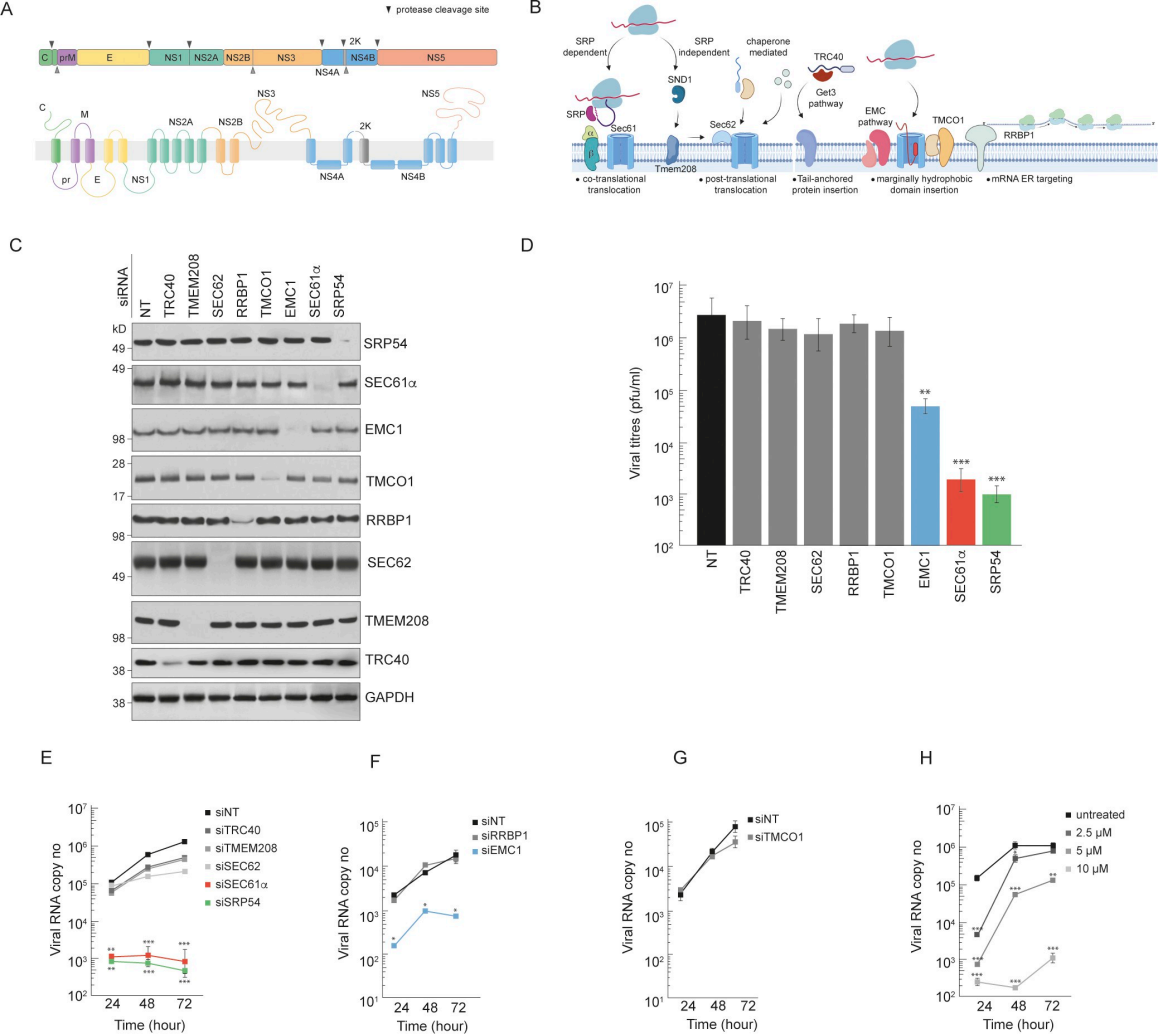
ZIKV polyprotein biogenesis requires the SRP-dependent co-translational ER translocation pathway. **(A)** Schematic of the ZIKV polyprotein showing the structural (C, prM, E) and non-structural (NS1-NS5) proteins, with transmembrane domains indicated. **(B)** Overview of the different ER-targeting pathways tested for involvement in ZIKV polyprotein synthesis, including the SRP-dependent co-translational translocation pathway (via Sec61 translocon), SRP-independent post-translational translocation (via Sec62 and Sec61), chaperone-mediated pathway, tail-anchored protein insertion (via TRC40 and TMCO1), mRNA targeting to ER (via RRBP1), and multi-pass transmembrane protein insertion (via EMC). Schematic was generated using Biorender. **(C)** Immunoblots validating siRNA-mediated depletion of factors from the ER targeting pathways in Huh7 cells, with GAPDH as loading control; NT is non-targeting control. Samples were collected 72 hours post-transfection with siRNAs to allow for optimal protein depletion. For TMCO1, samples were collected at 24 hours post-transfection. (**D**) Plaque assay quantification of ZIKV titres from supernatants of infected Huh7 cells at 48 hours post infection, following depletion of the indicated ER targeting factors. Data represent mean ± SD (n = 3). (**E-G**) Time course of infectious ZIKV production in HeLa cells depleted of indicated genes in comparison to siNT control. Viral copy numbers were determined by RT-qPCR quantification. Data represent mean ± SD (n = 3). (**H**) Dose-dependent inhibition of ZIKV production in Huh7 cells by Eeyarestatin I (ESI). Viral copy numbers were determined by RT-qPCR quantification at the indicated times post infection. Statistical significance was determined by two-tailed unpaired Student’s t-test, with * p < 0.05, ** p < 0.01, *** p < 0.001. (See also S1 Fig).

The positive-sense Zika viral RNA is first translated to a single polyprotein at the ER membrane, followed by its processing into 3 structural and 7 non-structural proteins (**Fig 1A**). Like all flaviviruses, the Zika virus polyprotein contains multiple signal sequences and transmembrane domains that facilitate their insertion into the ER membrane. The capsid protein contains a C-terminal signal sequence, while the prM and E proteins have transmembrane domains that serve as both stop-transfer and signal sequences for downstream proteins (**Fig 1A**) [36,37]. While flavivirus translation is believed to occur at the ER membrane, empirical data showing interaction of the signal sequences with the SRP is currently lacking. This model is supported by RNAi and CRISPR screens showing that SRP-translocon components are crucial for efficient virus propagation [28,29].

To confirm involvement of specific ER-targeting pathways (**Fig 1B**) in this process, we depleted components of the major mechanisms using siRNA transfection in Huh7 and HeLa cells (**Figs 1C and S1**). These included Sec61α, the core translocon subunit (**Figs 1C and S1A**); Sec62, the auxiliary subunit of the post-translational translocon [38] (**Figs 1C and S1B**); SRP54, the GTPase that recognises signal sequences in the SRP pathway (**Figs 1C and S1C**); TMEM208, the human homolog of Snd2 and proposed factor in the SND pathway [21,39] (**Figs 1C and S1D**); TRC40, which inserts tail-anchored proteins [40](**Figs 1C and S1E**); EMC1, the largest subunit in EMC complex for insertion of multi-pass proteins [22,23,41] (**Figs 1C and S1F**); RRBP1 (also known as p180), which promotes mRNA and ribosome ER localisation [42–44] (**Figs 1C and S1G**); and TMCO1, an Oxa1 homolog for hydrophobic domain insertion [40] (**Figs 1C and S1H**). Depletions reduced protein levels to <25% of control cells and were maintained for ≥ 72 hours (except TMCO1, where depletion could only be maintained up to 24 hours).

We infected the depleted cells with ZIKV at MOI 0.1, and quantified viral titres in Huh7 cell supernatants at 48h post infection by plaque assay (**Fig 1D**). We also measured viral RNA copy numbers over time in the supernatants of infected HeLa cells using RT qPCR (**Fig 1E–1G**). To rule out potential off-target effects, we used multiple independent siRNAs targeting the key proteins (**S1I Fig**). All siRNAs recapitulated the inhibition of viral replication observed with the primary siRNA (**Figs 1E–1G and S1J**), confirming the specificity of these effects. At least ~10–100 fold lower viral copies resulted from Sec61α, SRP54 or EMC1 depleted cells (**Fig 1D–1G**). A recent report indicated that EMC is required for insertion of the hydrophobic domains of NS4A and NS4B [31,32]. Our data indicates ZIKV production is partially EMC-dependent but primarily exploits the SRP and Sec61 translocon for polyprotein biogenesis.

To ensure that lack of phenotype with the other genes were not due to ineffective knockdown or compensatory mechanisms, we assessed the effects of siRNA-mediated depletion on known cellular substrates for each pathway. Depletion of TRC40 reduced levels of the tail-anchored protein cytochrome b5 [45], TMEM208 knockdown reduced the SND pathway substrate SGTA [21], RRBP1 depletion reduced secretion of procollagen-I [46], and TMCO1 depletion increased ER-targeted calcium levels [47] (**S1K Fig**). These results demonstrate that the siRNAs effectively disrupted their target pathways, despite not significantly impacting ZIKV replication.

To further validate reliance on co-translational translocation, we used Eeyarestatin I (ESI) and Mycolactone, pharmacological inhibitors that block signal sequence transfer from the SRP-RNC-SR complexes to the Sec61 [48–50]. Both ESI and Mycolactone treatments reduced ZIKV production in a dose-responsive manner (**Figs 1H and S1L**). These results demonstrate that ZIKV is reliant on the SRP-dependent ER targeting pathway for co-translational translocation.

### ZIKV translation and replication does not rely on the SR complex

**Fig 2:**
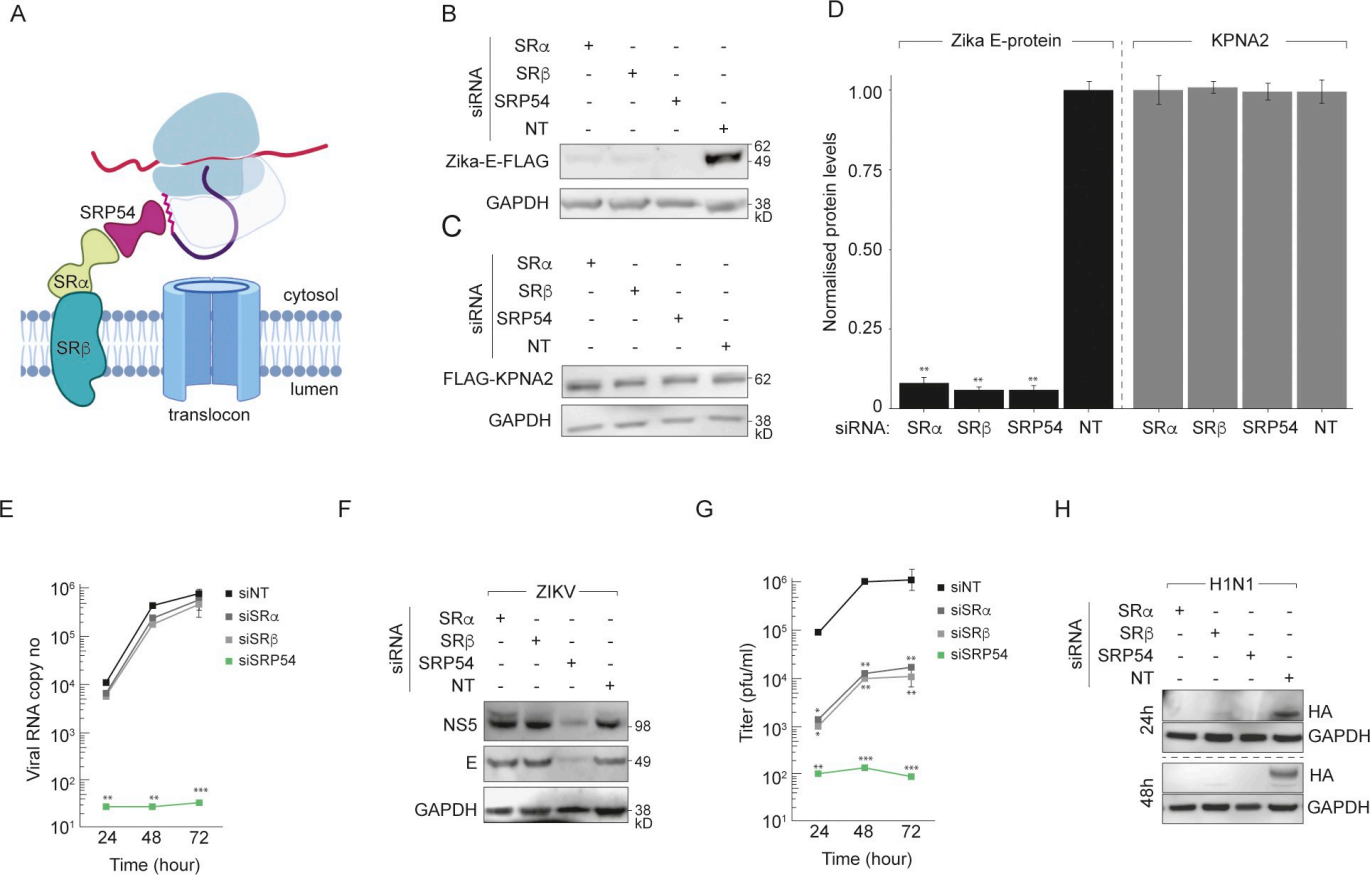
Zika virus polyprotein biogenesis is SRP54-dependent but SRα/SRβ-independent. **(A)** Schematic showing the interaction between the signal recognition particle (SRP) containing the SRP54 subunit and the SRP receptor (SR) composed of the SRα and SRβ subunits on the ER membranes. Schematic was generated using Biorender. **(B-C)** HeLa cells depleted of SRα, SRβ or SRP54 by siRNA were transfected with plasmids encoding (B) FLAG-tagged ZIKV prME protein or (C) FLAG-tagged KPNA2, a cytosolic protein, and analysed by immunoblotting. GAPDH served as loading control; siNT represents non-targeting control siRNA. **(D)** Quantification of relative ZIKV E and KPNA2 protein levels from (B-C). Bars represent mean ± SD (n = 3), with protein levels normalised to siNT control. **(E)** ZIKV growth kinetics in HeLa cells depleted of SRα, SRβ or SRP54. Viral RNA in supernatants was quantified by RT-qPCR. Data represent mean ± SD (n = 3). **(F)** Immunoblot analysis of ZIKV NS5 protein levels at 24 and 48 hours post infection (hpi) in HeLa cells depleted of SRα, SRβ or SRP54. GAPDH served as loading control. **(G)** Influenza A virus (H1N1 strain) growth kinetics in cells depleted of SRα, SRβ or SRP54. Viral titres in supernatants were measured by plaque assays on MDCK cells. Data represent mean ± SD (n = 3). **(H)** Immunoblot analysis of influenza hemagglutinin (HA) protein levels at 24 and 48 hpi cells depleted of SRα, SRβ or SRP54. GAPDH served as loading control. Statistical significance was determined by two-tailed unpaired Student’s t-test, with * p < 0.05, ** p < 0.01, *** p < 0.001. See also S2 Fig

To investigate which SRP components are necessary for ZIKV, we depleted the 3 GTPases in the SRP system—SRα, SRβ or SRP54 (**Fig 2A**)—in HeLa, Huh7 and Vero cells (**S2A–S2C Fig**). While CRISPR-Cas9 knock out of SR genes resulted in nonviable clones, depletion of SRα, SRβ or SRP54 by siRNA transfection did not significantly affect cell viability and survival. To confirm functional deficiency, we expressed plasmids encoding the soluble protein FLAG-KPNA2 (also known as importin α1) and the ZIKV membrane protein ZIKV-prME-FLAG (**Fig 2B and 2C**). As anticipated, FLAG-KPNA2 levels were not detectably reduced by depletion. However, intracellular ZIKV-E-FLAG levels were substantially lower in SRα, SRβ or SRP54 KD cells (**Fig 2B–2D**). These results confirm that depletion of either SRα, SRβ or SRP54 specifically inhibits membrane protein accumulation.

To verify the role of the SRP and SR in live virus infection, we infected the SRα, SRβ and SRP54 depleted HeLa cells with ZIKV at MOI 0.1. SRP54 depletion significantly reduced viral copy numbers in supernatants at all time points (**Fig 2E**). In contrast, minimal to no change occurred with SRα or SRβ loss. Similar results were obtained from Vero and Huh7 cells (**S2D and S2E Fig**), indicating these results are not cell type dependent or due to off-target effects. Moreover, while intracellular NS5 levels (the viral protein synthesised last in the polyprotein) decreased upon SRP54 knockdown, SRα or SRβ depletion did not detectably reduce NS5 levels (**Fig 2F**). Collectively, these results demonstrate ZIKV relies on SRP54 but not the SRP receptors SRα and SRβ for efficient propagation and membrane protein accumulation during infection.

To determine whether SRα/SRβ independent protein synthesis is unique to ZIKV or a common phenomenon in virus infection, we infected the knockdown cells with influenza A virus. Unlike flaviviruses, influenza replicates its RNA genome inside the nucleus and does not generate ER-resident non-structural proteins. The influenza virus surface protein hemagglutinin (HA) requires SRP-mediated ER targeting as a type I membrane protein. In line with ZIKV infection, SRP54 depletion significantly reduced influenza viral titres (**Fig 2G**). However, in contrast to ZIKV, SRα or SRβ loss also inhibited viral production, matched by substantially lower HA levels in all knockdown cells (**Fig 2H**). Hence, while ZIKV bypasses the SRP receptor requirement, influenza virus HA synthesis relies on SRα, SRβ, and SRP54. These results demonstrate ZIKV uniquely uncouples from dependence on the SRP receptor complex during viral translation, unlike influenza and potentially other viruses that lack ER-localised replicase proteins.

### Host membrane protein synthesis is altered upon ZIKV infection

**Fig 3:**
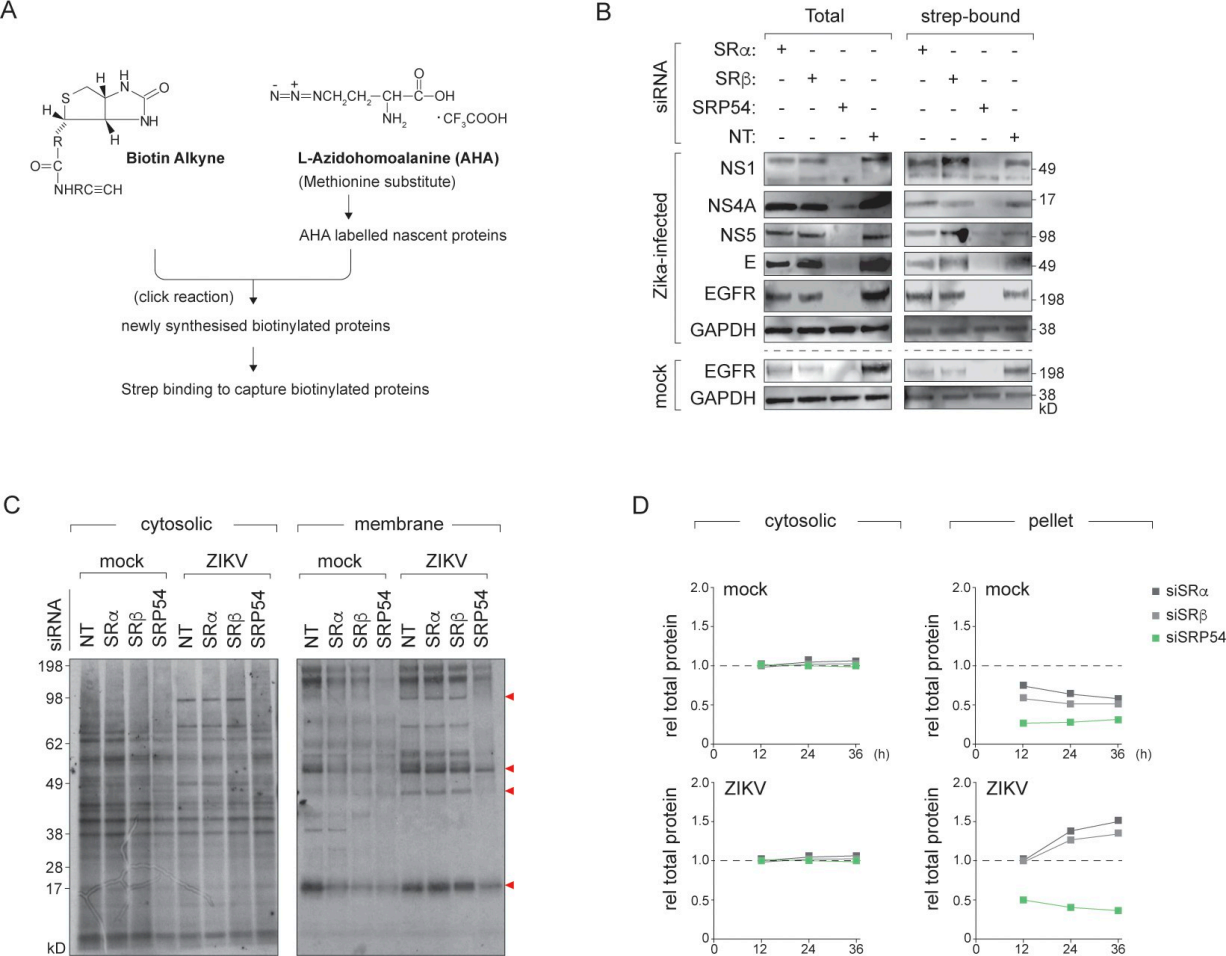
ZIKV infection alters host membrane protein synthesis in a SR-independent manner. **(A)** Workflow for quantifying nascent protein synthesis using L-azidohomoalanine (AHA) labelling. **(B)** Analysis of host and viral protein synthesis in ZIKV-infected HeLa cells depleted of SRα, SRβ or SRP54. Cells were pulsed with AHA for 4 hours at 24 hpi, and nascent proteins were captured on streptavidin beads and immunoblotted as described in (A). GAPDH and EGFR were probed as representative cytosolic and membrane proteins, respectively. **(C)** [^35^S]-labelled mock- or ZIKV-infected HeLa cells depleted of SRα or SRβ were separated into cytosolic and membrane fractions and analysed by autoradiography. Red arrowheads indicate proteins specifically inhibited in SRP54, but not SRα or SRβ-depleted cells. **(D)** Quantification of [^35^S]-labelled proteins in cytosolic and membrane fractions using scintillation counting. Graphs show relative total protein levels over time, normalised to the mock siNT control. Data represent mean ± SD (n = 3).

To quantify nascent protein synthesis, we pulsed cells depleted of SRα, SRβ or SRP54 with L-Azidohomoalanine (AHA), a clickable methionine analogue, in mock-treated or ZIKV-infected conditions. We normalised total protein in lysates, then conjugated alkyne biotin to AHA-labelled newly synthesised proteins. Streptavidin enrichment and immunoblotting was performed to analyse biotinylated translation products (**Fig 3A**). Gapdh and EGFR were used as representative models for translation of host cytosolic and membrane proteins, respectively.

Total Gapdh levels and newly synthesised Gapdh were comparable across conditions, suggesting unaffected cytosolic protein synthesis in SRα, SRβ or SRP54 depletion (**Fig 3B**). While EGFR levels decreased in all mock-infected knockdown cells (with the strongest phenotype in SRP54-depleted cells), the rate of EGFR synthesis in ZIKV-infected SRα or SRβ depleted cells was comparable to wild-type levels, despite lower total EGFR. This could indicate a partial rescue of EGFR synthesis during infection or changes in protein stability. However, we note that the effects are modest. Appropriately sized viral proteins were observed in SRα or SRβ depleted cells, indicating successful ER insertion and processing. Comparable viral protein synthesis levels between SR-depleted and the non-targeting control cells suggest viral polyprotein synthesis and stability remains unaffected without SRα or SRβ.

To further investigate whether ZIKV infection alters bulk membrane protein synthesis, we pulse labelled mock-infected or ZIKV-infected wild-type and SRα/SRβ depleted cells with [^35^S]cysteine/methionine, then fractionated the lysates into cytosolic and membrane fractions and visualised by autoradiography (**Fig 3C**) or quantitated by scintillation counting (**Fig 3D**). Cytosolic protein synthesis remained unaffected across conditions (**Fig 3C and 3D**). However, while membrane protein synthesis decreased substantially in mock-infected SRα/SRβ depleted cells, synthesis was rescued in ZIKV-infected cells, regardless of SR-depletion (**Fig 3C and 3D**). Membrane protein synthesis remained SRP54-dependent in both mock and virus-infected cells (**Fig 3D**). These results therefore suggest that upon infection, membrane protein biogenesis regulators are altered, conferring SR-independence. Collectively, SR loss impairs membrane protein production in uninfected cells, but ZIKV infection uniquely modifies and enhances ER-localised translation in an SR-independent manner without affecting cytosolic protein output.

### ZIKV non-structural proteins recruit the SRP-RNC complex to the ER

**Fig 4:**
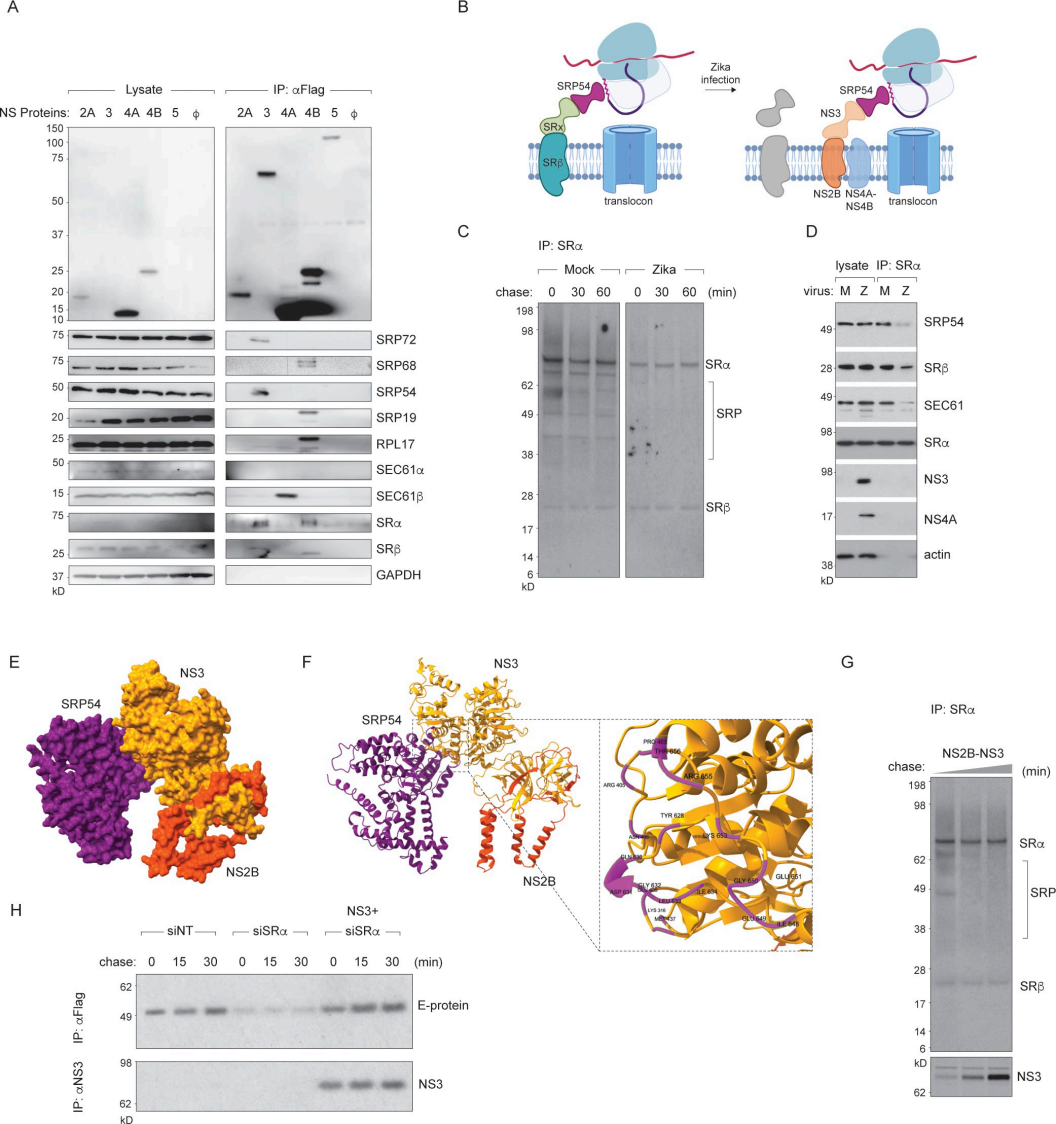
ZIKV non-structural proteins interact with SRP and the translocon to facilitate ER targeting. **(A)** FLAG-tagged ZIKV non-structural proteins NS2A, NS2B-NS3, NS4A, NS4B, and NS5 were expressed in HeLa cells and immunoprecipitated using anti-FLAG antibodies. Lysates and co-precipitating proteins were analysed by immunoblotting. GAPDH served as a loading control in lysates. **(B)** Schematic representation of altered interaction of SRP with the viral non-structural proteins in virus infected cells. Schematic was generated using Biorender. **(C)** Mock- or ZIKV-infected HeLa cells were metabolically labelled with [^35^S]-cysteine/methionine for 30 min and chased for the indicated times. Endogenous SRα was immunoprecipitated and co-precipitating proteins were analysed by autoradiography. **(D)** Immunoblot analysis of SRα immunoprecipitates from mock (M) and virus-infected (Z) cells. **(E, F)** Structural models of SRP54 interactions with ZIKV NS2B-NS3 (E) generated using AlphaFold. The SRP54 NG domain forms a key interface (F, inset) for binding to the viral proteins. **(G)** HeLa cells ectopically expressing inducible viral NS2B-NS3 were labelled with [^35^S]-cysteine/methionine. Lysates were immunoprecipitated with anti-SRα and co-precipitating proteins were analysed by autoradiography. **(H)** Control or SRα depleted HeLa cells were transfected with prME-FLAG and NS2B-NS3 plasmids, metabolically labelled with [^35^S]-cysteine/methionine and chased for the indicated times. E-protein and NS3 were immunoprecipitated on anti-FLAG and anti-NS3 antibodies and visualised by autoradiography. See also S3 Fig.

We hypothesised that SR requirement during virus polyprotein biogenesis might be bypassed on account of SRP interacting directly with the virus proteins at the ER. To gain insights into potential interactions between ZIKV non-structural proteins and the host translational machinery, we performed co-immunoprecipitation using C-terminally Flag-tagged NS2A, NS2B/NS3, NS4A, 2k/NS4B and NS5 expressed in HeLa cells and probed for associated endogenous factors (**Fig 4A**). We detected interactions between various viral proteins and SRP components, including viral NS3 binding to SRP54 and SRP72; NS4A binding to the Sec61β translocon subunit; and NS4B binding to SRP19, SRP68 and the ribosome large subunit Rpl17 (**Fig 4B**).

To control for potential cross-reactions, we performed siRNA depletions of SRP components and before performing anti-FLAG immunoprecipitations. These controls confirmed the specificity of the observed interactions (**S3 Fig**). Notably, SRP54 depletion reduced expression of NS3 and NS4B, likely due to the role of SRP54 in membrane protein biogenesis. The double band observed for NS4B (**Fig 4A**) is due to partial processing of the 2K peptide in the expression construct, which is more evident in the enriched IP samples compared to total lysates. Additionally, the variability in the pull-down of SRP54 by NS4B between experiments, may reflect the dynamic nature of these interactions or indirect interactions. Similarly, the low expression levels of NS3 make it difficult to detect in lysates but becomes visible after IP enrichment (**Fig 4A**).

To investigate potential interactions between viral non-structural proteins and host translational machinery components in infected cells, we performed immunoprecipitation experiments using [^35^S]cysteine/methionine-labelled virus-infected cells (**Fig 4C and 4D**). Our data indicate that the interaction of SRα with newly synthesised proteins is dramatically altered between uninfected versus virus infected cells. While SRα/SRβ clearly interacts with the SRP in mock infected cells, this interaction is impaired upon infection. Instead, we observed interactions between various viral non-structural proteins, including NS3 and NS4B, with SRP components, along with increased interaction with the translocon complex (**Fig 4C and 4D**).

To investigate the interaction between SRP54 and viral non-structural proteins, we performed both structural predictions using Alphafold, and experimental validation through immunoprecipitation. In line with our observations with immunoprecipitations from infected samples, NS2B-NS3 in particular was predicted to interact with the SRP, with the NG domain forming a key interface in this interaction (**Fig 4E and 4F**). To further validate these findings, we ectopically expressed viral NS2B-NS3 in uninfected cells and performed immunoprecipitation of SRα. Our results demonstrate that the interaction between SRα and SRP subunits was reduced with increasing concentrations of NS2B-NS3 (**Fig 4G**), supporting our prediction that it can compete with SRα for binding to SRP54. In addition, overexpression of NS2B-NS3 in SRα-depleted cells restored prME-FLAG expression to wild-type levels (**Fig 4H**), further supporting this hypothesis.

**Fig 5:**
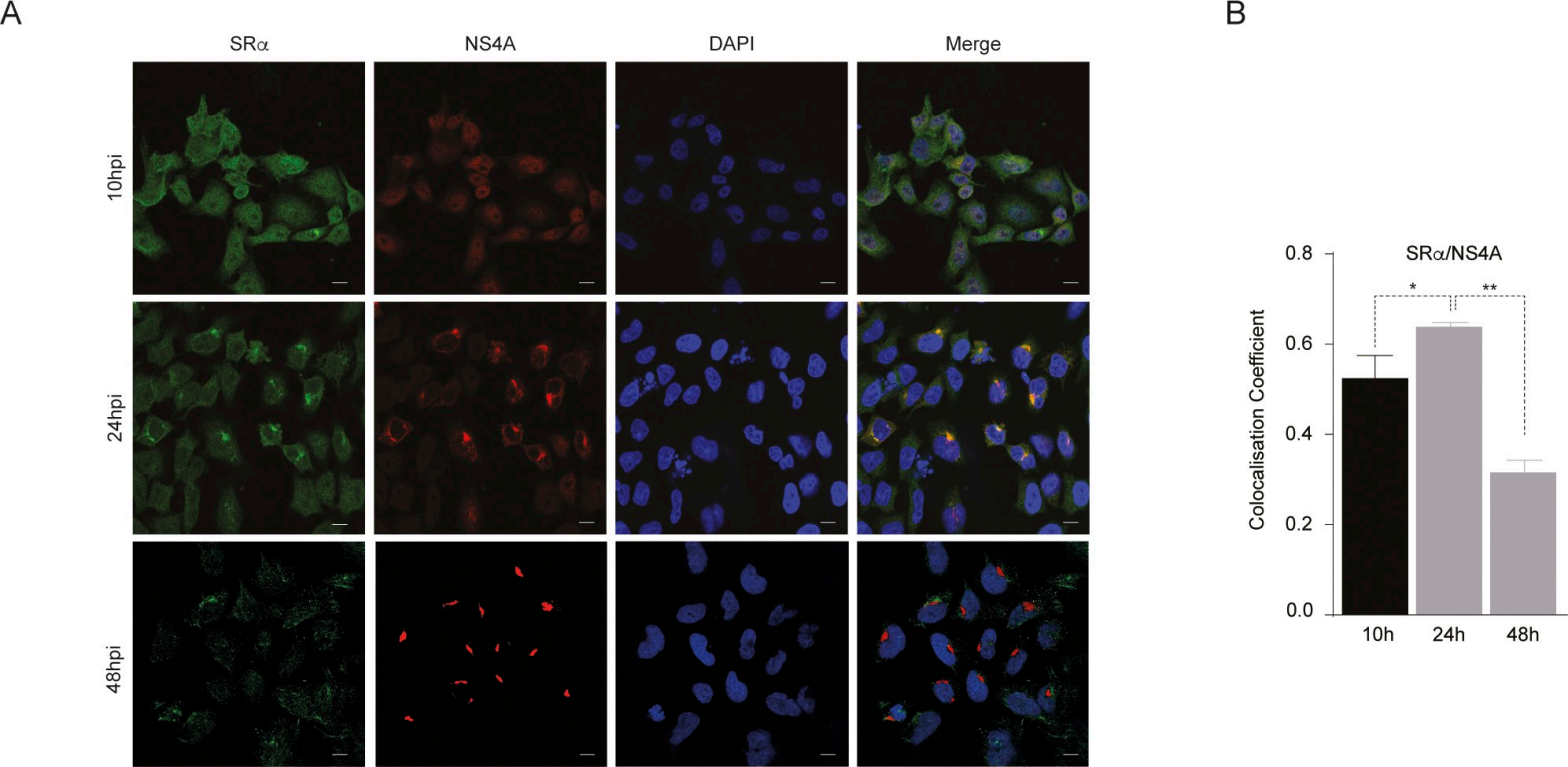
Time course of SRα and NS4A localisation during ZIKV infection. **(A)** Immunofluorescence analysis of SRα (green) and ZIKV NS4A (red) localisation in HeLa cells at 10, 24, and 48 hours post-infection (hpi) with ZIKV. Nuclei were stained with DAPI (blue). At 10 hpi, both SRα and NS4A were diffusely distributed throughout the cell, with no apparent co-localisation. At 24 hpi, NS4A began to form distinct puncta, likely representing viral replication complexes, which partially co-localised with SRα. By 48 hpi, NS4A puncta became more prominent and numerous, while SRα co-localisation decreased. Scale bar, 10 μm. **(B)** Quantification of the co-localisation coefficient between SRα and NS4A at different time points post-infection. The co-localisation coefficient was calculated using Pearson’s correlation and represents the fraction of SRα that colocalises with NS4A. Data represent mean ± SEM (n = 30 cells per time point), with * p < 0.05 and ** p < 0.01 by one-way ANOVA with Tukey’s post hoc test.

To determine whether SR components are present at replication sites during infection, we performed confocal imaging in ZIKV infected HeLa cells stained for SRα (representing the SR complex) and NS4A (representing the replication complexes) (**Fig 5**). At 10 hour-post-infection, NS4A and SRα were evenly dispersed in cells without any obvious co-localisation. At 24 hours post infection, NS4A clusters indicative of replication complexes, were found to colocalise with SRα. At 48 hours post infection, increase in NS4A clusters were observed with subsequent decrease in co-localisation with SRα, suggesting that SRP receptors were excluded from sites of viral polyprotein synthesis at later timepoints in the viral lifecycle (**Fig 5A and 5B**).

Collectively, these data imply that interaction of SRα with SRP54 via the NG-domain may be replaced by the viral non-structural proteins potentially for more efficient synthesis of the viral and selected host proteins during infection.

### Virus replicase complex proteins serve as receptors for SRP targeting to the ER in infected cells

**Fig 6:**
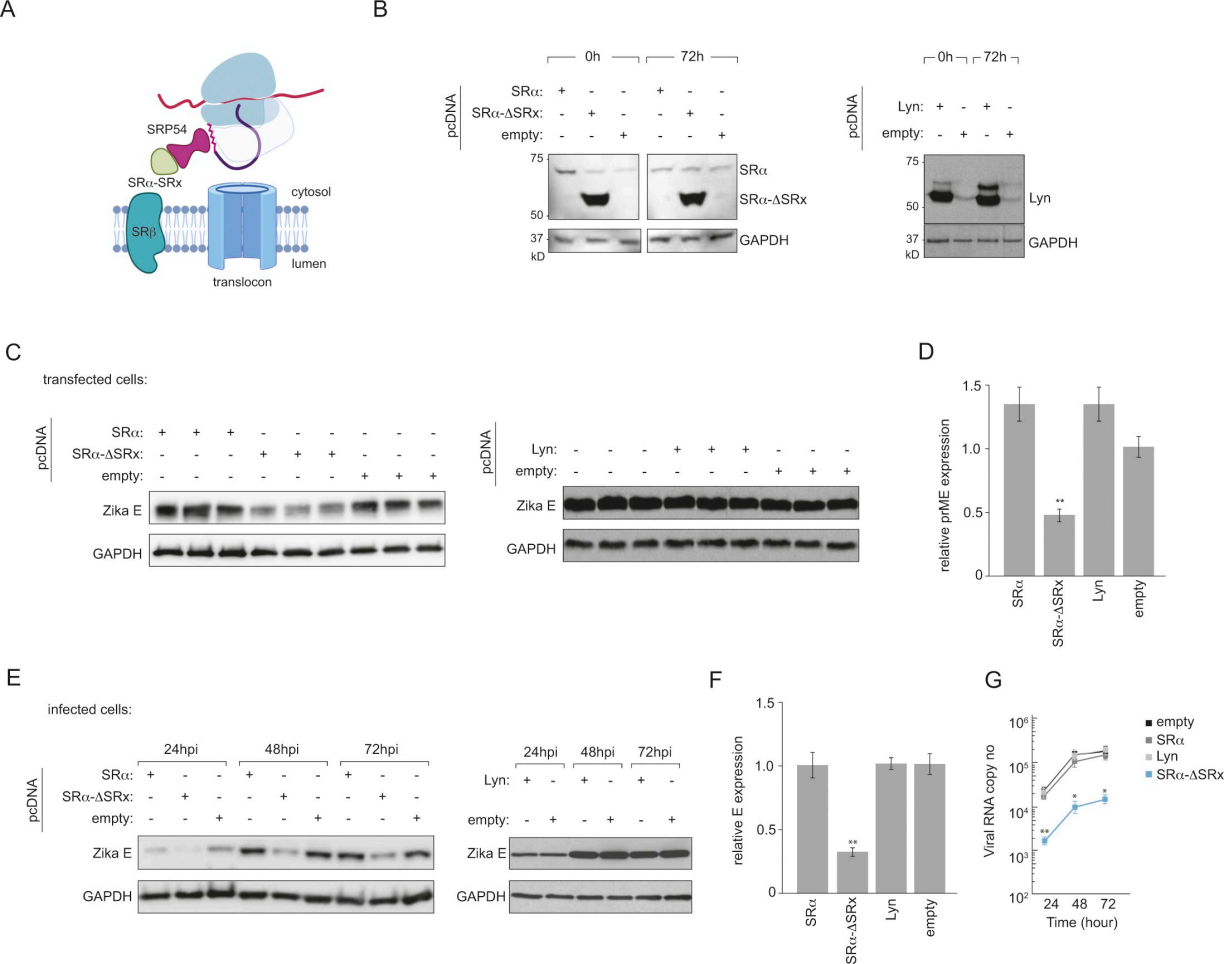
Overexpression of SRα-ΔSRx inhibits ZIKV polyprotein synthesis and viral replication. **(A)** Schematic of the proposed model for ZIKV non-structural proteins bypassing SRα/SRβ by directly interacting with SRP54 to facilitate ER targeting of the viral polyprotein. Overexpression of the SRP54-interacting domain of SRα (SRα-ΔSRx) is hypothesised to competitively inhibit the interaction between viral proteins and SRP54. Schematic was generated using Biorender. **(B)** Immunoblot analysis of SRα-ΔSR_X_ and Lyn kinase expression in HeLa cells transfected with empty vector, SRα, SRα-ΔSR_X_ and Lyn kinase plasmids. GAPDH served as a loading control. **(C-D)** Inhibition of ectopic prME-FLAG expression by SRα-ΔSR_X_. HeLa cells transfected with empty vector, SRα-ΔSR_X_ or Lyn-kinase were transfected with a plasmid encoding ZIKV prME protein. (C) Immunoblot analysis and (D) quantification of relative prME levels, normalised to empty vector control. Data represent mean ± SD (n = 3). **(E-F)** Inhibition of ZIKV protein synthesis by SRα-ΔSR_X_. HeLa cells transfected with empty vector, SRα, SRα-ΔSR_X_ or Lyn kinase were infected with ZIKV (MOI = 1). (E) Immunoblot analysis of ZIKV E protein levels at the indicated timepoints. (F) Quantification of relative E protein levels at 72 hpi, normalised to empty vector control. Data represent mean ± SD (n = 3). **(G)** ZIKV growth kinetics in HeLa cells transfected with empty vector, SRα-ΔSR_X_ or Lyn kinase. Supernatants were collected at the indicated timepoints, and viral RNA was quantified by RT-qPCR. Data represent mean ± SD (n = 3). Statistical significance was determined by two-tailed unpaired Student’s t-test, with * p < 0.05, ** p < 0.01.

Based on our findings, we hypothesised that virus non-structural proteins such as NS2B3 directly interact with SRP to circumvent the need for SRα/β and facilitate ER targeting during infection (**Fig 6A**).

SRα is composed of two domains—an N-terminal domain that interacts with SRβ (SRx domain) and a C-terminal domain that interacts with the NG domain of SRP54. We therefore hypothesised that if either SRα or the soluble SRα component that binds SRP54 (SRα-ΔSR_X_) is overexpressed in these cells, this would competitively inhibit binding of SRP to the non-structural proteins and thereby inhibit virus protein synthesis, and by extension virus production. We generated a truncated version of SR lacking the SR_X_ domain (SRα-ΔSR_X_) that binds to SRP, but is not targeted to the ER (**Fig 6B**). We reasoned that when overexpressed, SRα-ΔSRx would compete with viral proteins for SRP54 binding, potentially inhibiting virus replication. While over-expression of the full-length SRα could not be achieved, SRα-ΔSR_X_ expressed to high levels (**Fig 6B**). To control for potential non-specific effects of protein overexpression, we also expressed Lyn kinase, a cytosolic protein of similar size to SRα-ΔSRx but unrelated to the SRP pathway. We first assessed potential non-specific effects of SRα-ΔSR_X_ expression on cell viability. MTT assays showed no significant difference in cell viability between SRα-ΔSR_X_-expressing cells and the control (**S4 Fig**). Both SRα-ΔSRx and Lyn kinase expressed at comparable levels (**Fig 6B**).

We first measured general membrane protein biogenesis by ectopic expression of Zika prME. Cells expressing SRα-ΔSR_X_ showed significantly reduced prME levels compared to empty vector or Lyn-kinase expressing cells (**Fig 6C and 6D**). This suggests that SRα-ΔSR_X_ specifically interferes with the expression of membrane proteins.

We next investigated the impact of SRα-ΔSR_X_ on ZIKV infection. HeLa cells expressing empty vector, SRα-ΔSR_X_, or Lyn kinase were infected with ZIKV (MOI = 1). Intracellular levels of ZIKV E protein were significantly lower in SRα-ΔSR_X_-expressing cells at all time points post-infection, while Lyn kinase expression had no effect (**Fig 6E and 6F**). To assess functional consequences of SRα-ΔSR_X_ expression on viral replication, we measured ZIKV RNA levels in the supernatants of infected cells over time. Consistent with the reduced viral protein levels, SRα-ΔSR_X_-expressing cells produced significantly less viral RNA compared to control cells or those expressing Lyn kinase (**Fig 6G**). Collectively, these data support our model that ZIKV relies on direct interactions between viral proteins and SRP54 for efficient replication, bypassing the need for the SRP receptor. The specific inhibition by SRα-ΔSR_X_, but not by an unrelated protein (Lyn kinase) expressed at similar levels, suggests that competitive inhibition of SRP54 binding to viral proteins is sufficient to impair viral replication. However, it is important to note that our current data do not distinguish between [1] inhibition of SRP54-viral protein interactions at later stages of infection, or [2] reduced polyprotein insertion at early times when canonical SRP-SR pathways are still operational.

### ER-specific remodelling of protein synthesis occurs in ZIKV-infected cells

**Fig 7:**
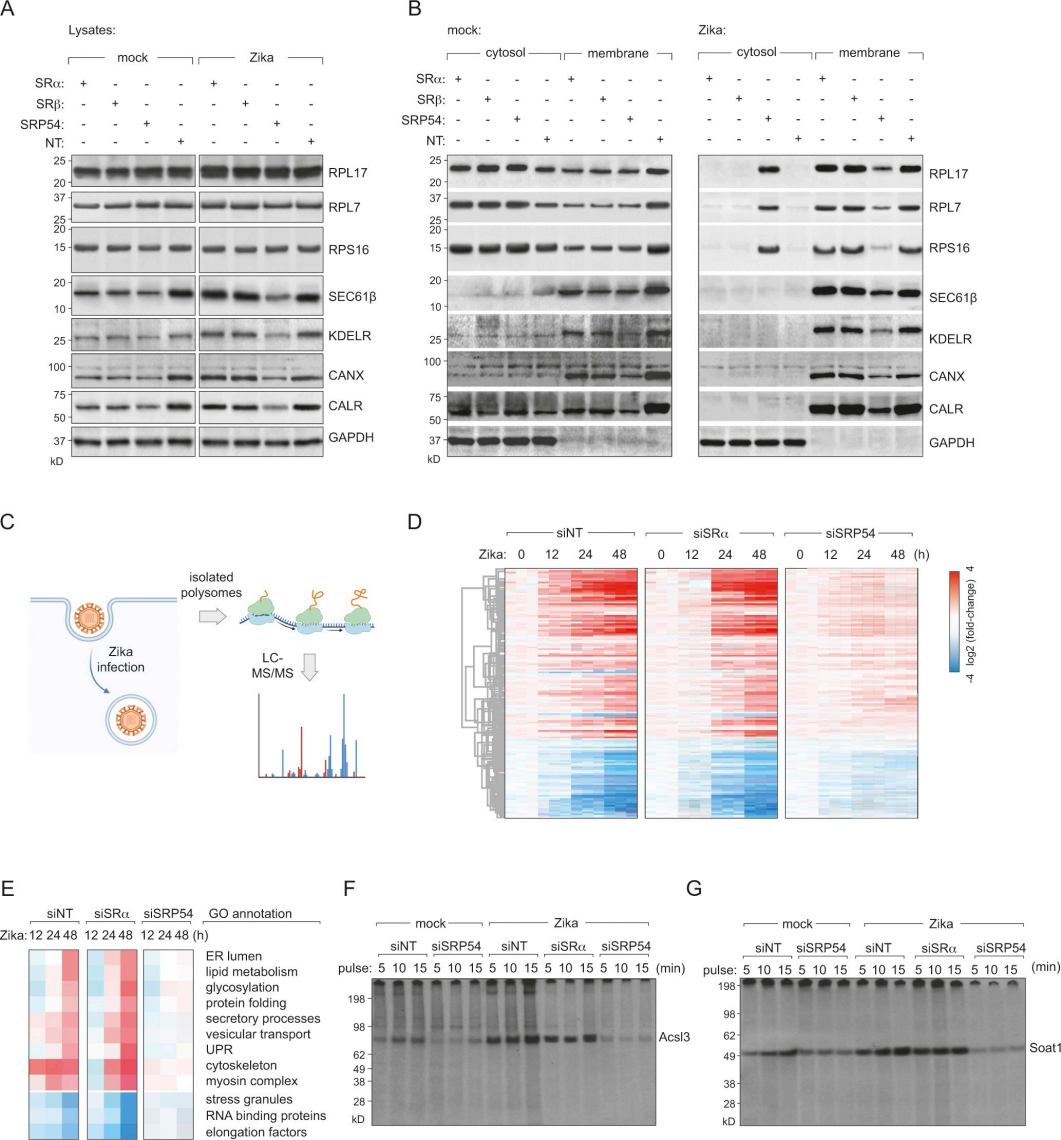
ZIKV infection remodels the ER translatome in a SR-independent manner. **(A, B)** Mock- or ZIKV-infected cells depleted of SRα, SRβ, or SRP54 were fractionated into cytosolic and membrane fractions, and the distribution of marker proteins was analysed by immunoblotting. (A) Immunoblots of markers in lysates (B) Immunoblots of cytosolic and membrane fractions from mock- or ZIKV-infected cells. GAPDH served as a cytosolic marker, while KDEL receptor (KDELR), calnexin (CANX), and calreticulin (CALR) were used as ER markers. RPL17 was probed to assess ribosome association with ER membranes. **(C)** Schematic of the polysome profiling and mass spectrometry experiment. Polysomes were isolated from mock- or ZIKV-infected HeLa cells depleted of SRα, SRβ, or SRP54 at different timepoints post-infection. Polysome-associated proteins were identified and quantified by LC-MS/MS. Schematic was generated using Biorender. **(D-E)** Heatmaps showing the relative abundance of polysome-associated proteins **(E)** ZIKV infection led to an enrichment of proteins involved in ER membrane expansion, lipid metabolism, and secretory processes, while proteins associated with RNA binding and stress granules were depleted. **(F-G)** Validation of polysome profiling results by [^35^S]-cysteine/methionine labelling. Mock- or ZIKV-infected HeLa cells depleted of SRα or SRP54 were pulse-labelled for the indicated times, and newly synthesized proteins were analysed by autoradiography. **(F)** ER-associated proteins ACSL3 (lipid metabolism) and **(G)** SOAT1 (cholesterol esterification) showing increased synthesis in ZIKV-infected cells. Images are representative of at least 2 independent experiments.

Our results suggest that recruitment of polysomes to the ER would remain unaffected in the absence of SRα/β in Zika virus infected cells. To specifically measure recruitment of ribosomes to the ER, we quantified cytosolic and ER-associated ribosomes. Mock and virus-infected cells were fractionated in the presence of cycloheximide and RNase inhibitors to preserve ribosome-RNA interactions (**Fig 7A and 7B**). This was performed in WT cells as well as in those depleted in either SRP54, SRα or SRβ. While Gapdh was present solely in the cytosolic fraction, the ER proteins KDELR, calnexin and Sec61β were found in the membrane fractions. In uninfected cells, although total levels of ribosomal large and small subunits (Rpl17, Rpl7, Rps16) were comparable across all conditions, their ER-associated levels were substantially lower in SRα, SRβ or SRP54 depleted cells (**Fig 7B**). The fraction of ER-associated ribosomal subunits was substantially higher in Zika-infected cells compared to that of the mock infected controls. While SRP54 knockdown led to reduced ER-associated ribosomal proteins in infected cells, loss of SRα or SRβ did not affect their ER-association (**Fig 7B**). The comparable levels of ribosomal subunits recruited to the ER in wild-type and SRα or SRβ-deficient cells following Zika infection, suggest that while SRP plays a significant role in viral polyprotein biogenesis, SRα and SRβ components are dispensable in infected cells. Collectively, these data indicate that the interaction of SRP with viral non-structural proteins potentially enables subversion of ER translation from host transcripts to viral transcripts, in line with previous reports on increased dengue RNA localisation to the ER [51].

To systematically assess how this remodelling might impact synthesis rates of host proteins in virus-infected cells, we isolated actively translating polysomes from wild-type, SRP54-depleted or SR-depleted cells at different timepoints post infection and identified the associated proteins by mass spectrometry (**Fig 7C**). This approach allowed us to identify both newly synthesised proteins and ribosome-associated factors. We performed unsupervised hierarchical clustering on the proteins that showed significant differential association with polysomes across our experimental conditions (ANOVA, FDR < 0.01). This analysis revealed distinct temporal patterns of protein enrichment and depletion in the polysome fractions during the course of ZIKV infection. Importantly, these dynamic changes in polysome association were not mirrored in the total cellular proteome, suggesting that ZIKV infection induces specific alterations in the translational landscape rather than global changes in protein abundance. Specific host proteins involved in ER expansion and lipid metabolism were enriched over time in infected cells, while those associated with RNA binding and stress granule formation were depleted as previously reported [52] (**Fig 7D, 7E and S1 Table**). Interestingly, we observed an initial decrease at early timepoints of infection (12h post infection) in SR-deficient cells, followed by recovery at later stages (24h and 48h post infection) (**Fig 7D, 7E and S1 Table**). These findings were validated in metabolically labelled cells (24 h post infection), which displayed infection-specific alterations in protein synthesis, such as increased synthesis of lipid metabolic enzymes (e.g. ACSL3) and cholesterol esterification (SOAT1) compared to uninfected cells (**Fig 7F and 7G**). These data suggest that initial rounds of ER targeting (prior to virus non-structural protein synthesis) relies on the SRP-SR interaction. However, once viral non-structural proteins are produced, SRP-viral protein binding substitutes for SRP-SR, enabling the retention of translating polysomes at the ER membrane.

**Fig 8:**
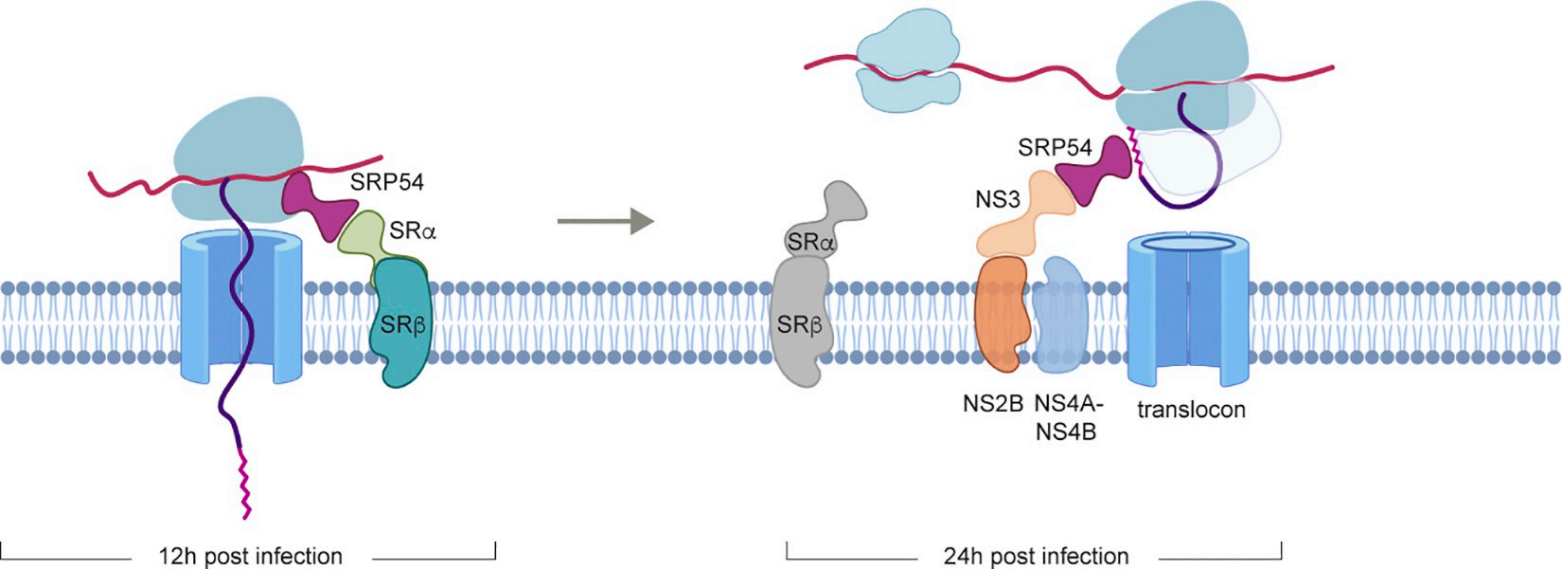
Proposed model for ZIKV-mediated remodelling of ER targeting and altered translatome. During infection, ZIKV non-structural proteins (NS2B-NS3, NS4A, NS4B) interact with SRP54 and the translocon to bypass the requirement for SRα/SRβ and facilitate ER targeting of the viral polyprotein. This leads to selective enrichment of host factors involved in ER membrane expansion, lipid metabolism, and secretory processes at the translating ribosomes, while RNA-binding proteins and stress granule components are excluded. This translational reprogramming supports viral replication and suppresses antiviral responses. The viral non-structural proteins play a key role in this process by hijacking the SRP-dependent targeting pathway and recruiting host factors necessary for viral replication to the ER. Schematic was generated using Biorender.

To further corroborate our results from polysome profiling results and determine whether Zika virus infection selectively inhibits stress granule formation, we quantified G3BP1-positive puncta in cells exposed to poly (I:C), 3p-shRNA, UV-inactivated or live Zika virus (**S5A–S5D Fig**). While cells stimulated with the various stress-inducers displayed an accumulation of G3BP1 positive granules, Zika-infected cells exhibited a significantly reduced abundance of these structures (**S5C and S5D Fig**). These findings demonstrate that active ZIKV replication induces significant changes in the profile of newly synthesised host proteins. Collectively, our data demonstrate that flavivirus infection is accompanied by specific remodelling of the endoplasmic reticulum, facilitating efficient synthesis of viral and host proteins necessary for replication, assisted by the viral non-structural proteins (**Fig 8**).

## Discussion

In this study, we uncovered a mechanism by which Zika virus remodels the SRP-dependent co-translational translocation pathway to ensure efficient polyprotein synthesis and virus replication. Our findings reveal that while Zika virus relies on SRP54 for its biogenesis, it has evolved a unique strategy to bypass the requirement for the SRP receptors, SRα or SRβ, which are typically essential components of the SRP pathway. This is achieved through interactions between viral non-structural proteins and various components of the SRP and translational machinery, though the precise nature and specificity of these interactions require further investigation. This is in contrast to other viruses, such as influenza, which depends on both SRP and SR subunits for its translation and replication, and display distinct strategies of ER remodelling from flaviviruses [53].

Through a series of biochemical experiments, we identified an extensive network of interactions between Zika viral non-structural proteins and key host factors involved in protein synthesis and translocation, including SRP, ribosomes and the translocon. Remarkably, even in the absence of SRα or SRβ, Zika infection resulted in an increased association of ribosomes with the ER, and enhanced membrane protein synthesis, suggesting that the virus can efficiently recruit and concentrate these factors at the translation/replication sites, bypassing the need for SR subunits. Structural modelling using Alphafold further supported our findings, indicating that Zika non-structural proteins can act as surrogate SRP receptors. These viral proteins can bridge the SRP-RNC complexes with the translocon, facilitating targeting and insertion of the viral polyprotein into the ER membrane.

The ability of Zika virus to bypass the SR subunits and directly recruit SRP and ribosomes to the ER membrane has important implications for viral replication strategies. By concentrating ribosomes and associated viral RNAs at the ER, the virus increases the likelihood of initiating translation on ER-associated ribosomes, reducing the dependence on ER targeting pathways such as SRP, SND and TRC40. This spatial regulation of viral translation and replication can enhance the efficiency of polyprotein synthesis and processing, as nascent polypeptides can be directly inserted into the ER membrane via the translocon. The accumulation of viral proteins and RNA in the SR-depleted cells further supports this hypothesis. However, depletion of SRP54 significantly impairs virus biogenesis, likely due to its critical role in recognising and shielding hydrophobic signal sequences as they emerge from the ribosome exit tunnel. In the absence of SRP54, these hydrophobic domains may be exposed to the aqueous cytosolic environment, resulting in protein misfolding, aggregation, and the induction of the unfolded protein response (UPR) that can hinder virus replication.

It is worth noting that despite the potential importance of SRα/β in the early stages of infection, our depletion experiments did not show a significant effect on overall virus replication. This apparent discrepancy can be explained by several factors. Firstly, both SRα and SRβ are non-essential genes, and protein synthesis can occur in their absence [54], albeit at a reduced rate. Our study employed siRNA-mediated depletion rather than complete gene knockout, which likely resulted in residual levels of SRα/β. These limited amounts may have been sufficient to support initial viral protein synthesis, which would have been more severely impaired in cells completely lacking SRα and SRβ. Once the viral non-structural proteins accumulate to sufficient levels, they may compensate for the reduced SRα/β levels by directly interacting with SRP components and the translocon, as our data suggest.

Our findings raise intriguing questions about the evolutionary adaptation of flaviviruses and their ability to manipulate host cellular pathways. The interaction between viral proteins and host protein synthesis machinery may have implications for pathogenesis of Zika and other flaviviruses. By modulating the ER translatome and concentrating SRP and ribosomes at replication sites, the virus may alter the balance of host protein synthesis, potentially contributing to virus survival and cellular dysfunction. Our study therefore reveals a sophisticated mechanism by which Zika virus rewires the SRP-dependent translocation pathway, providing insights into the complex virus-host interplay.

## Materials and Methods

### Cell lines

HeLa (ATCC CCL-2), Vero (ATCC CCL-81), Huh7 (JCRB Cell Bank JCRB0403), A549 (ATCC CCL-185), MDCK (ATCC CCL-34) and C6/36 cells (ATCC CRL-1660) were maintained in Dulbecco’s Modified Eagle Medium (DMEM) (Gibco) with 10% fetal bovine serum (FBS) (Gibco). HeLa, Vero, Huh7, A549 and MDCK cells were kept at 37°C, where C6/36 cells were kept at 25°C, all with 5% CO_2_. Cells were passaged sub-confluently with 0.25% Trypsin-EDTA (Gibco).

### Plasmids

The prM and E genes of dengue virus were amplified by PCR from viral cDNA [13] using primers: Forward: 5′-CCCGAATTCTGAACGGGAGAAAAAGGT-3′ and Reverse: 5′-GGGGGTACCATTCTGCTTGAACTGTGAAGC-3′. The PCR product was digested with EcoRI and NotI and cloned into pcDNA3.1(+) vector (Invitrogen). The KPNA2 gene was amplified from HeLa cell cDNA using primers: Forward: 5’-ATGGCGGACAAAGCCGAGAG-3’ and Reverse: 5’-TCAGGGACAGCTTGCTGTTG-3’. The PCR product was digested with BamHI and NotI and cloned into pcDNA3.1(+) vector. The inducible lentiviral NS2B-NS3 used in this study has been described previously [12].

### Dicer-Substrate (DsiRNA) transfection

DsiRNAs were designed using a custom DsiRNA design tool and purchased from Integrated DNA technology (IDT). The sequences of the DsiRNA used are summarised in S2 Table. 10pmol of the DsiRNAs were transfected to 24-well HeLa, A549, Vero or Huh7 cells (2x10^4^ cells/well) using Lipofectamine RNAiMAX Transfection Reagent (Invitrogen) following the manufacturer’s recommended protocol and concentration. Cells were incubated at 37°C with 5% CO_2_ for 72 hours before further experiments were performed.

### Virus infections

#### Zika Virus infection and propagation

The Asian Strain ZIKV, NC-5132-2014, was propagated in C6/36 cells at 25°C with 5% CO2 for 18 hours. Viral stocks were titrated using plaque assay with Vero cells. HeLa, Vero or Huh7 cells transfected with DsiRNA were infected with ZIKV at 0.1 multiplicity of infection (MOI) for 1 hour in serum free DMEM. Cells were washed twice with PBS and then incubated in DMEM containing 2% FBS at 37°C with 5% CO2. Cell supernatants were collected at the indicated time points for viral quantification by one-step reverse transcribed-quantitative polymerase chain reaction (RT-qPCR), using primer pairs as used previously [55] with sequences 5’-AGGATCATAGGTGATGAAGAAAAGT-3’ and 5’-CCTGACAACACTAAGATTGGTGC-3’; or plaque assay with Vero cells using methylcellulose overlay. List of other primers are listed in S3 Table.

#### Influenza A Virus infection and propagation

Influenza A Virus, A/Puerto Rico/8/1934 (PR8), was propagated in MDCK cells in serum free DMEM supplemented with 1μg/mL N-tosyl-L-phenylalanine chloromethyl ketone treated Trypsin (TPCK-Trypsin) (Sigma Aldrich) at 37°C with 5% CO_2_ for 72 hours. Viral stocks were titrated using plaque assays from MDCK infected cells. After transfecting with DsiRNA, A549 cells were infected with PR8 at 0.1 MOI for 1 hour in serum free DMEM. Cells were washed twice with PBS and then incubated in serum free DMEM supplemented with 0.2μg/mL TPCK-Trypsin at 37°C with 5% CO_2_. Cell supernatants were collected at the indicated time points and titrated with plaque assays on MDCK cells with agarose overlay.

### Transfections and luciferase assays

HeLa cells were transfected with the indicated plasmids for 48 hours using Lipofectamine 2000 (Invitrogen) according to the manufacturer’s protocol. For pathway-specific reporter assays, cells were co-transfected with the respective luciferase reporter plasmids 24 hours after siRNA transfection. 24 hours after reporter construct transfection, culture medium was removed, and cells were washed once with PBS. 100 μL of 1X Passive Lysis Buffer (Promega) was added to each well, and the plate was incubated at room temperature for 15 minutes with gentle shaking. Luciferase activity was measured using the Dual-Luciferase Reporter Assay System (Promega) according to the manufacturer’s instructions. For the TMCO1-dependent calcium regulation assay, cells were treated with ionomycin immediately before measurement to induce calcium influx.

### Immunoprecipitation and immunoblotting

Viral non-structural genes from the Zika virus Asian strain SZ01, were cloned into the pcDNA3.1(+) plasmid (Invitrogen) with an added FLAG-tag at C-termini using primers summarised in S3 Table. The plasmids were transfected into HeLa cells and incubated for 48 hours. Cells were lysed by 25G syringe strokes in IP-buffer (150mM NaCl, 50mM Tris pH 8.0, 1% digitonin or Triton X-100) with *cOmplete Protease Inhibitor (Roche)*. Cell lysates were cleared by centrifugation at 20,000 x g for 10 minutes at 4°C. Cleared lysates were incubated with Anti-FLAG-M2 Affinity Agarose Gel (Sigma), with rotation at 4°C for 2 hours. FLAG-tagged proteins and its interactors were eluted by NuPAGE LDS Sample Buffer (Invitrogen) at 95°C after washing with PBS. Eluent and cell lysates were analysed by immunoblot. Protein samples were analysed by homemade SDS-PAGE or 4–12% Bis-Tris NuPage (Invitrogen).

### Quantification of nascent protein synthesis

HeLa cells transfected with specific DsiRNA were infected with ZIKV at MOI 1 for 24 hours. Cells were starved in methionine-free DMEM (Invitrogen) before pulsing with 50μM L-Azidohomoalanine (AHA) (Invitrogen) for 4 hours. Cells were lysed in lysis buffer (50 mM NaCl, 50 mM Tris pH 8.0, 1.0% Triton X-100, 0.5% sodium deoxycholate, 0.1% sodium dodecyl sulphate). The amount of protein in cell lysates was normalised after quantification using BCA assay (Pierce). Biotin was conjugated to AHA residues by adding a click reaction mix (40 μM alkyne biotin (Invitrogen), 2 mM sodium ascorbate (Sigma), 100 μM tris[(1-benzyl-1H-1,2,3-triazol-4-yl)methyl]amine (TBTA) (Sigma) and 2 mM copper(II) sulphate (Sigma) at final concentration). The reaction mix was rotated end-over-end overnight. Unreacted alkyne biotin was removed by 7K MWCO Zeba spin desalting columns (Invitrogen). Biotinylated proteins were enriched using streptavidin-conjugated magnetic beads (Invitrogen) and analysed by Immunoblotting.

#### Metabolic labelling for nascent protein synthesis

Metabolic labelling experiments were performed to analyse newly synthesised proteins and their interactions as previously described [12,53]. HeLa cells were either mock-infected or infected with ZIKV at an MOI of 1. At 24 hours post-infection, cells were prepared for pulse-chase analysis. Cells were detached using 0.25% trypsin-EDTA and washed twice with PBS. The cell pellet was resuspended in methionine/cysteine-free DMEM and incubated at 37°C for 30 minutes to deplete intracellular methionine and cysteine pools. Cells were labelled with 10 mCi [^35^S]methionine/cysteine (PerkinElmer, expressed protein labelling mix) for indicated times at 37°C, and chased in complete DMEM containing an excess of unlabelled methionine/cysteine. Cell pellets were lysed in ice-cold lysis buffer (50 mM Tris-HCl pH 7.4, 150 mM NaCl, 1% Triton X-100, 1 mM EDTA) supplemented with protease inhibitor cocktail. Lysates were centrifuged at 14,000 x g for 10 minutes at 4°C to remove cellular debris. Pre-cleared lysates were incubated with anti-SRα or anti-FLAG antibodies (2 μg per sample) for 3 hours at 4°C with gentle agitation.

Antibodies anti-Sec61α (Invitrogen, PA5-21773), anti-Sec61β (Abcam, ab229542), anti-Sec62 (Abcam, ab140644), anti-TMEM208 (Abcam, ab130459), anti-TRC40 (Abcam, ab169539), anti-SRP19 (Abcam, ab131239), anti-SRP54 (Abcam, ab154796), anti-SRP68 (Invitrogen, PA5-100080), anti-SRP72 (Abcam, ab200199), anti-EGFR (Abcam, ab52894), anti-SRβ (Abcam, ab236725), anti-SRα (Abnova, H00006734-B02P), anti-RPL17 (Abcam, ab155781), Anti-Zika virus NS4A (GeneTex, GTX133704), Anti-Influenza A Virus Hemagglutinin (Abcam, ab139361), Anti-Zika virus Envelope (homemade 4G2 mouse monoclonal antibody from supernatants of a hybridoma clone and GeneTex, GTX133314), anti-TMCO1 (Invitrogen, PA5-43350) and anti-EMC1 (Invitrogen, PA5-31597). Goat anti-Mouse IgG (H+L) Secondary Antibody, HRP (Invitrogen) and Goat anti-Rabbit IgG (H+L) Secondary Antibody, HRP (Invitrogen) were used as secondary. Antibodies were used in concentrations suggested by the suppliers. All quantification of immunoblot were performed using Fiji.

### Immunofluorescence Microscopy

HeLa cells seeded on glass cover slides were infected with ZIKV at MOI 4. Cells were incubated in a 37°C incubator with 5% CO2 for 10, 24 or 48 hours before fixation with 4% formaldehyde/PBS. Cells were permeabilised with 0.1% Triton X-100/PBS and stained with mouse anti-SRα (Abnova, H00006734-B02P) and rabbit anti-NS4A (GeneTex, GTX133704). Cover slides were stained with Goat anti-mouse IgG Alexa Fluor 488 (Invitrogen) and anti-rabbit IgG Alexa Fluor 555 (Invitrogen). Cell nuclei were counterstained with 4′,6-diamidino-2-phenylindole (DAPI). Cover slips were mounted on glass slides and viewed on a Carl Zeiss LSM780 microscope.

### Cell Fractionation

Cells were ZIKV or mock infected at MOI 1 for 24 hours. Cell fractionation was performed with a modified protocol from [45]. To preserve RNA interaction with ribosomes, 1mM DTT, 180 μM cycloheximide (Cell Signalling), 1× Complete Protease Inhibitor Cocktail (Roche) and 40 U/ml RNaseOUT (Invitrogen) were freshly added to all reagents used for fractionation. Briefly, cells were scraped and lysed in cytosol buffer (50 mM NaCl, 50 mM Tris pH 8.0, 0.03% Digitonin) with 10 passages through a 25-G needle. Lysates were spun and supernatants collected as the ‘cytosol fraction’. Pellets were washed twice in a wash buffer (50 mM NaCl, 50 mM Tris pH 8.0, 0.015% Digitonin) before lysing the pellet with ER buffer (50mM NaCl, 50mM Tris pH 8.0, 2% n-dodecyl-β-D-maltoside (Invitrogen). Lysates were spun again and the supernatants collected as the ‘ER fraction’. Protein levels in the cytosolic and ER fractions were normalised before analysis by immunoblots.

### Polysome profiling and mass spectrometry

For polysome profiling, mock- or ZIKV-infected HeLa cells (MOI 1, 12, 24 and 48 hours post-infection) were treated with cycloheximide (100 μg/mL) for 10 minutes prior to harvesting. Cells were lysed in polysome buffer (20 mM HEPES pH 7.6, 100 mM KCl, 5 mM MgCl2, 1% Triton X-100, 100 μg/mL cycloheximide, 1x protease inhibitor cocktail, 100 U/mL RNase inhibitor) and fractionated on 10–50% sucrose gradients by ultracentrifugation at 36,000 rpm (SW41 Ti rotor) for 2.5 hours at 4°C. Fractions corresponding to polysomes (254 nm) were pooled and proteins were precipitated using methanol/chloroform extraction. Protein samples were reduced, alkylated, and digested with trypsin overnight. Resulting peptides were desalted using C18 StageTips and analysed on a Q Exactive HF mass spectrometer (Thermo Fisher) coupled to an EASY-nLC 1200 UHPLC system (Thermo Fisher). Peptides were separated on a 25 cm reversed-phase column (75 μm inner diameter, 1.9 μm ReproSil-Pur C18-AQ resin) over a 120-minute gradient. MS data were analysed using MaxQuant (version 1.6.10.43) and searched against the human UniProt database and ZIKV polyprotein sequence. Label-free quantification was performed using the MaxLFQ algorithm. Statistical analysis was done in Perseus (version 1.6.12.0). Proteins with valid values in at least 3 out of 4 replicates in at least one condition were considered for analysis. Missing values were imputed from a normal distribution. Differentially enriched proteins were determined using a two-sample t-test with a permutation-based FDR of 0.05 and s0 of 0.1. Gene ontology enrichment analysis was performed using the Ingenuity Pathway Analyses software. Raw mass spectrometry data were deposited to the ProteomeXchange Consortium via the PRIDE partner repository with the dataset identifier PXD045790.

## Supporting information

S1 Fig Validation of siRNA-mediated knockdown of ER-targeting pathway components in HeLa cells. (PDF)

S2 Fig siRNA-mediated knockdown of SRP54, SRα, and SRβ in Huh7 and Vero cells and its impact on ZIKV replication. (PDF)

S3 Fig Validation of specificity of viral protein interactions with SRP54. (PDF)

S4 Fig Cell viability assay of HeLa cells expressing SRα-ΔSRx or Lyn kinase. (PDF)

S5 Fig ZIKV infection inhibits stress granule formation. (PDF)

S1 Table Enrichment of differentially synthesised proteins. (XLSX)

S2 Table List of DsiRNA used in this study. (XLSX)

S3 Table List of primers used in this study. (XLSX)
