# Supplementary material for: Flaviviruses induce ER-specific remodelling of protein synthesis
Source: PLoS Pathog. 2024 Dec 2;20(12):e1012766. doi: 10.1371/journal.ppat.1012766 (PMC11637433; doi:10.1371/journal.ppat.1012766)
Supplement: S1 Fig — (PDF) [file ppat.1012766.s001.pdf]

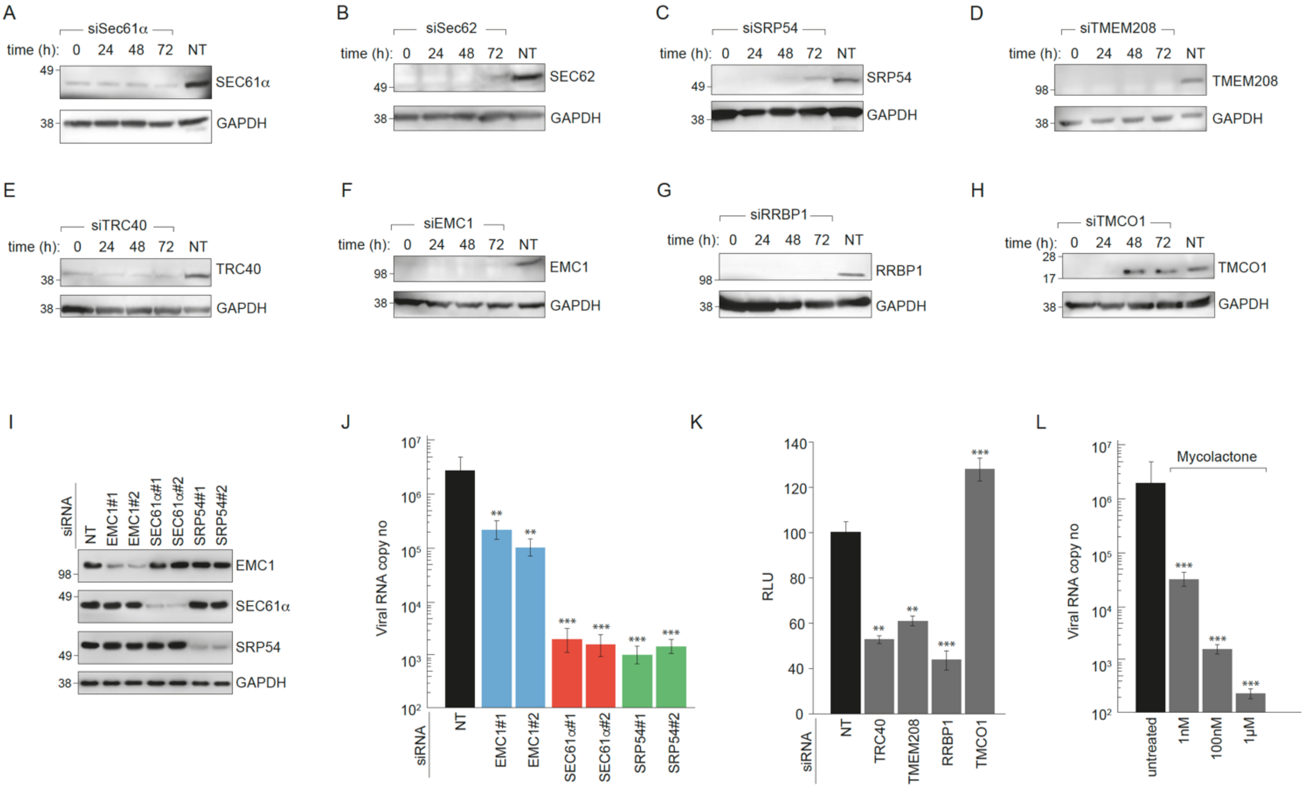

Figure S1. Validation of siRNA-mediated knockdown of ER-targeting pathway components in HeLa cells.

(A-H) Immunoblot analysis of protein levels following siRNA-mediated depletion of (A) Sec61α, (B) Sec62, (C) SRP54, (D) TMEM208, (E) TRC40, (F) EMC1, (G) RRBP1, and (H) TMCO1 in HeLa cells over a 72-hour time course. Cells were transfected with either non-targeting (NT) control siRNA or siRNA targeting the indicated ER-targeting pathway component. GAPDH served as a loading control. **(I)** Immunoblots showing knockdown efficiency of multiple independent siRNAs targeting EMC1, SEC61α, and SRP54 in HeLa cells. **(J)** RT-qPCR quantification of ZIKV RNA copy numbers in HeLa cells depleted with multiple independent siRNAs targeting EMC1, SEC61α, and SRP54. Data represent mean ± SD (n=3). **(K)** Functional validation of siRNA knockdowns in cells expressing reporter constructs of the indicated proteins. TRC40 depletion reduced cytochrome b5 levels, TMEM208 knockdown reduced SGTA, RRBP1 depletion reduced procollagen-I secretion, and TMCO1 depletion increased ER calcium levels as measured by relative luminescence units (RLU). **(L)** Dose-dependent inhibition of ZIKV production in Huh7 cells by Mycolactone. Viral RNA copy numbers were determined by RT-qPCR. Statistical significance was determined by two-tailed unpaired Student's t-test, with * $p < 0.05$, ** $p < 0.01$, *** $p < 0.001$.
