# Supplementary material for: Flaviviruses induce ER-specific remodelling of protein synthesis
Source: PLoS Pathog. 2024 Dec 2;20(12):e1012766. doi: 10.1371/journal.ppat.1012766 (PMC11637433; doi:10.1371/journal.ppat.1012766)
Supplement: S2 Fig — (PDF) [file ppat.1012766.s002.pdf]

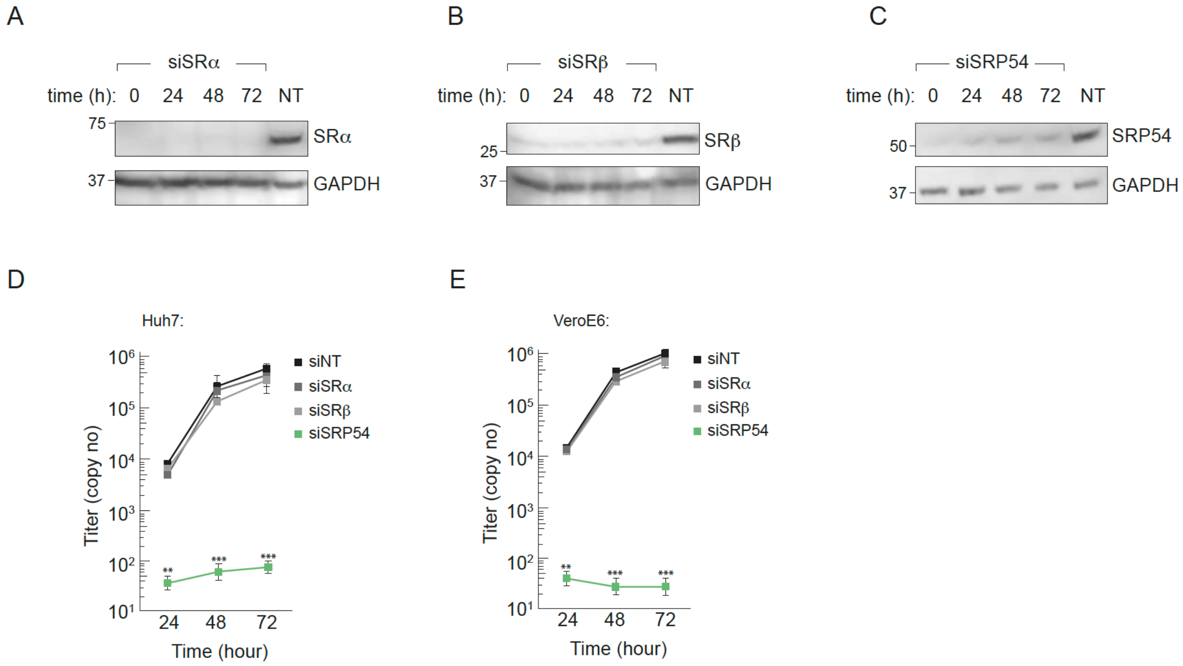

Figure S2. siRNA-mediated knockdown of SRP54, SRα, and SRβ in Huh7 and Vero cells and its impact on ZIKV replication.

(A-C) Immunoblot analysis of protein levels following siRNA-mediated depletion of (A) SRP54, (B) SRα, and (C) SRβ in Huh7 cells over a 72-hour time course. Cells were transfected with either non-targeting (NT) control siRNA or siRNA targeting the indicated protein. GAPDH served as a loading control. **(D-E)** ZIKV growth kinetics in (D) Huh7 and (E) Vero cells depleted of SRP54, SRα, or SRβ. Cells were transfected with the indicated siRNAs and infected with ZIKV at an MOI of 0.1. Viral RNA in the supernatants was quantified by RT-qPCR at 24, 48, and 72 hours post-infection. Data represent mean ± SEM (n=3), with ** $p < 0.01$ and *** $p < 0.001$ by two-tailed unpaired Student's t-test.
