# Supplementary material for: Flaviviruses induce ER-specific remodelling of protein synthesis
Source: PLoS Pathog. 2024 Dec 2;20(12):e1012766. doi: 10.1371/journal.ppat.1012766 (PMC11637433; doi:10.1371/journal.ppat.1012766)
Supplement: S3 Fig — (PDF) [file ppat.1012766.s003.pdf]

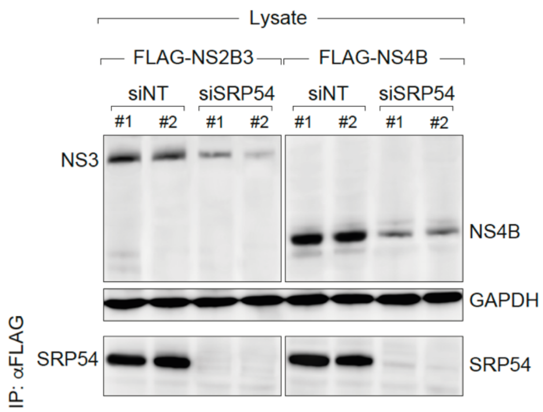


**Figure S3. Validation of specificity of viral protein interactions with SRP54.**

(Upper panel) HeLa cells were transfected with control or SRP54 siRNA for 48 hours, followed by transfection with FLAG-tagged NS2B3 or NS4B plasmids for 24 hours. Lysates were analysed by immunoblotting.

(Lower panel) Anti-FLAG immunoprecipitation from cells showing no cross-reacting bands in SRP54 depleted cells.
