# Supplementary material for: Flaviviruses induce ER-specific remodelling of protein synthesis
Source: PLoS Pathog. 2024 Dec 2;20(12):e1012766. doi: 10.1371/journal.ppat.1012766 (PMC11637433; doi:10.1371/journal.ppat.1012766)
Supplement: S4 Fig — (PDF) [file ppat.1012766.s004.pdf]

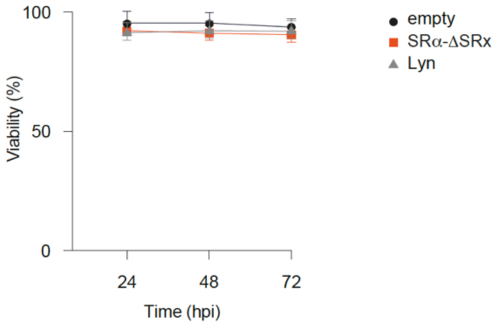

Figure S4. Cell viability assay of HeLa cells expressing SRα-ΔSRx or Lyn kinase.

HeLa cells were transfected with empty vector, SRα-ΔSRx, or Lyn plasmids. Cell viability was assessed using MTT assay at 24, 48, and 72 hours post-transfection. Data are presented as percentage of viable cells relative to empty vector control. Values represent mean ± SD from three independent experiments.
