# Supplementary material for: Flaviviruses induce ER-specific remodelling of protein synthesis
Source: PLoS Pathog. 2024 Dec 2;20(12):e1012766. doi: 10.1371/journal.ppat.1012766 (PMC11637433; doi:10.1371/journal.ppat.1012766)
Supplement: S5 Fig — (PDF) [file ppat.1012766.s005.pdf]

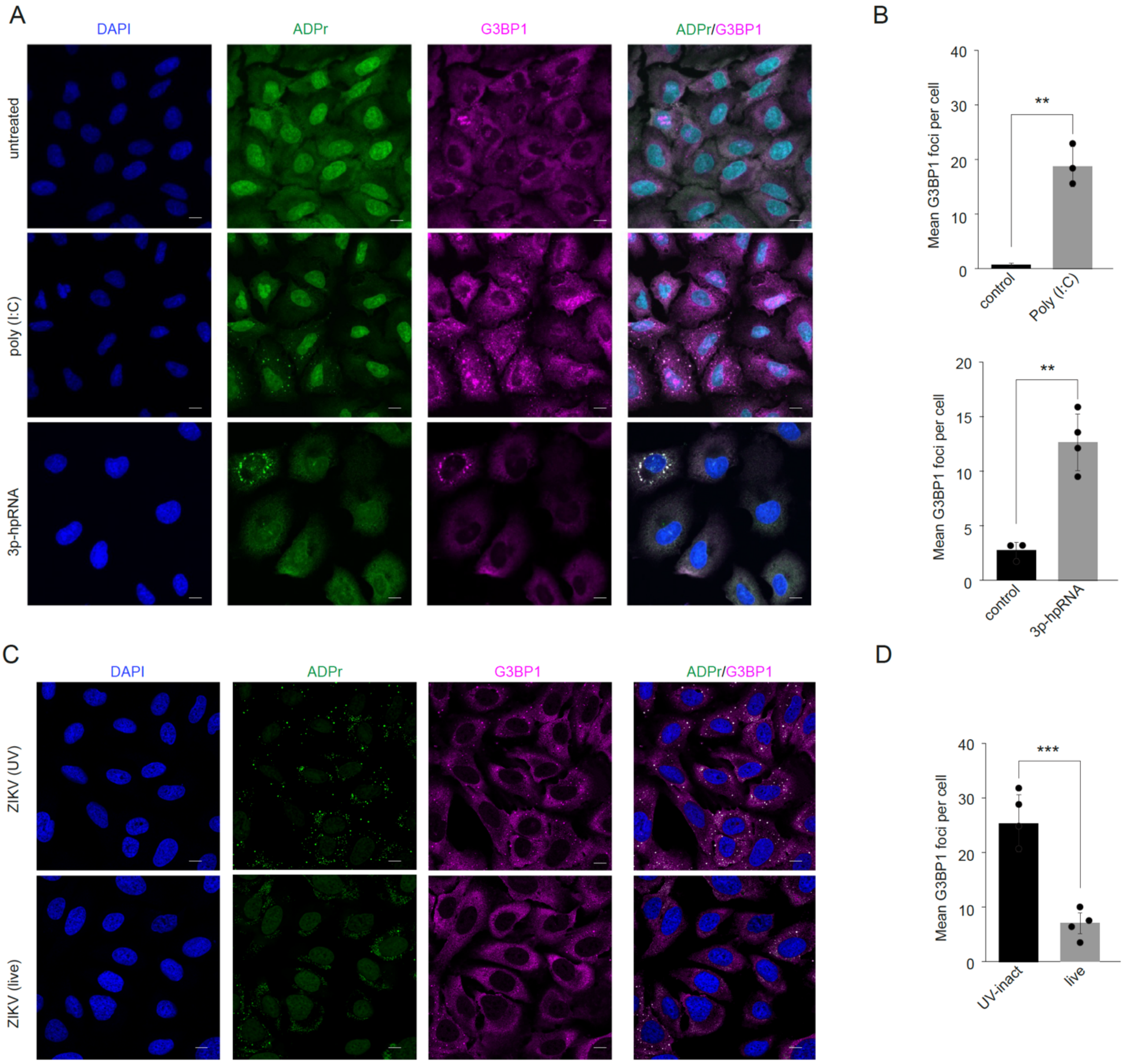

Figure S5. ZIKV infection inhibits stress granule formation

(A, C) Immunofluorescence images of mock- or ZIKV-infected cells stained for the stress granule marker G3BP1 (magenta), pan ADP-ribosylation (ADPr, green) and nuclei (DAPI, blue). Cells were either left untreated (mock), or transfected with (poly I:C), 3p-hpRNA, or infected with either UV-inactivated or live ZIKV. Scale bar, 10 µm. **(B, D)** Quantification of the mean G3BP1-positive puncta per cell in samples described in (A and C). Data represent mean ± SD (n=3), with ** $p < 0.01$ by two-tailed unpaired Student's t-test.
